# Very low prevalence of anti-HAV in Japan: high potential for future outbreak

**DOI:** 10.1038/s41598-018-37349-1

**Published:** 2019-02-06

**Authors:** Chikako Yamamoto, Ko Ko, Shintaro Nagashima, Takayuki Harakawa, Toshiko Fujii, Masayuki Ohisa, Keiko Katayama, Kazuaki Takahashi, Hiroaki Okamoto, Junko Tanaka

**Affiliations:** 1 Department of Epidemiology, Infectious Disease Control and Prevention, Graduate School of Biomedical and Health Sciences, Hiroshima University, Hiroshima, Japan; 2 Hiroshima Community Health Promotion Organization, Hiroshima Prefecture, Hiroshima, Japan; 3 Division of Virology, Department of Infection and Immunity, Jichi Medical University, School of Medicine, Tochigi, Japan; 4 Department of Medical Sciences, Tokyo Shinagawa Hospital, Tokyo, Japan

## Abstract

Since the early 21^st^ century, almost all developed countries have had a very low hepatitis A virus antibody (anti-HAV) sero-prevalence profile, as sanitation conditions and health care facilities have been optimized to a universal standard. There has not been a report on anti-HAV prevalence among a large scale population in Japan since 2003. Therefore, this study aimed to investigate the current HAV status among the general population in Hiroshima. From each age and sex specific group, a total of 1,200 samples were randomly selected from 7,682 stocked serum samples from residents’ and employees’ annual health check-ups during 2013–2015. Total anti-HAV was detected using Chemiluminescent Enzyme Immunoassay. The overall anti-HAV sero-prevalence was 16.8%. In both males and females, anti-HAV prevalence among individuals between 20–59 years of age was as low as 0.0–2.0%, whilst that among 70 s was as high as 70.0–71.0%. A large number of residents aged under 60 are now susceptible to HAV infection. The cohort reduction trend of anti-HAV in Japan exposes the high possibility of mass outbreak in the future. HAV vaccine especially to younger generation and high risk population may prevent outbreak in Japan.

## Introduction

Hepatitis A virus (HAV) infection occurs sporadically and is primarily transmitted via the fecal-oral route, bearing a high potential for either cyclic recurrence or explosive worldwide spread as an epidemic, especially in the case of a food or waterborne outbreak^1^. In addition, sexual transmission, especially in men who have sex with men have been documented^2^. However, HAV endemics are strongly related to socio-economic conditions, and such infections can be reduced by improving the hygiene, sanitary habits, and water supply of the population and by using HAV vaccination.

It has been estimated that millions people worldwide are infected with HAV each year. In 2015, there were approximately 11,000 deaths from HAV, contributing to 0.8% of the total death from viral hepatitis^3,4^. Although vaccination against HAV infection has been available since the early 1990s, it is not widely used^5,6^ and most people maintain immunity via exposure resulting from a childhood infection.

The severity of HAV infection greatly depends on the age at the time of viral entry. Approximately 90% of infections were asymptomatic among infected children under 5 years of age, whilst approximately 70% of infections cause the typical symptoms of acute hepatitis among older children and adults, of which less than 1% may progress into fatal fulminant hepatitis^7^. The severity of disease increases with age; more than 53% of adults ≥60 years old require hospitalization for acute hepatitis^8^.

HAV is a self-limiting disease that can resolve without inducing chronic infection or other manifestations. Individuals experiencing HAV infection with or without symptoms have lifelong immunity; in contrast, immunization through inactivated or live attenuated HAV vaccines does not guarantee lifelong immunity^9^. With a high proportion of the population not immune to HAV, deterioration in existing sanitation and water supply could lead to a massive transmission of HAV.

HAV endemicity levels vary worldwide, and regions are separated into three main categories: high, intermediate, and low endemic areas. These three regions indirectly indicate the socioeconomic level, including the sanitation, hygiene, and water supply of the country. In highly endemic countries, more than 90% of children have been exposed to HAV infection by 10 years of age, while ≥50% have seroconverted into anti-HAV positive by 15 years of age in intermediate countries and by 30 years of age in low endemic countries^10^. These three categories (high endemic areas, intermediate endemic areas, and low endemic areas) are determined based on whether the positive rate of anti-HAV IgG in human serum in the study population is <15%, 15–50%, or >50%^11^.

The National Institute of Infectious Disease in Japan conducted nationwide sero-surveys on HAV prevalence among the general population four times, in 1973, 1984, 1994, and 2003. Using these large scale nationwide surveys, the overall anti-HAV prevalence was reported to be 8% (1973), 10% (1984), 19.4% (1994), and 12.2% (2003). All studies revealed very low anti-HAV prevalence among the young population and a gradual increase in anti-HAV positivity after 50 years of age. Moreover, ≥10 year shift in anti-HAV prevalence in each age group was also found between the studies, showing persistent very low anti-HAV prevalence among the general population, especially in young adults under 50 years of age^12–14^.

After 2003, no more reports on anti-HAV prevalence among general population have been documented in Japan. The very low prevalence previously reported may threaten possible mass transmission of HAV in Japan. Therefore, it is important to know the current situation of HAV infection among the general population in Japan. We conducted this study to investigate the prevalence of anti-HAV among the general population in Hiroshima. Thereafter, the trend of HAV infection can be predicted more accurately and can be used for determining an effective strategy and action plan in Japan.

## Results

Of the total 1,200 serum samples (600 males and 600 females), 202 samples were anti-HAV positive, of which half of the samples were males. Hence, the overall anti-HAV prevalence was 16.8% [95% confidence interval (95%CI): 14.7–19.0%]. Sex had no effect on anti-HAV prevalence (16.8% each for both males and females).

**Figure 1:**
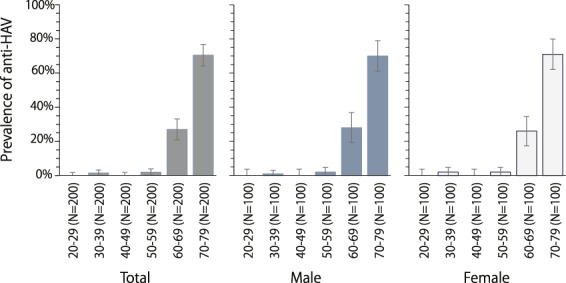
Sex and age specific prevalence of anti-HAV. The first grey color bar chart represents the overall anti-HAV prevalence of the 1,200 selected samples in each particular age group. The middle blue color bar chart represents the prevalence of age specific anti-HAV in males and the last white color bar chart represents females.

By subgroup analysis, the trend of anti-HAV prevalence in both males and females in each age specific group coincided with the overall prevalence of the same age group. The prevalence of anti-HAV in age groups 20–29, 40–49, and 50–59 were 0.0%, 0.0%, and 2.0%, respectively in both males and females. The prevalence of anti-HAV in age group 30–39 was 1.0% for males and 2.0% for females, respectively. This prevalence tended to increase to 28% and 70% for males and 26% and 71% for females of age groups 60–69 and 70–79, respectively (Fig. 1).

Anti-HAV sero-prevalence was extremely low in the younger generation but increased with age, with a cohort reduction trend of ≥10 year interval.

The estimated prevalence of anti-HAV positive individuals was nearly 1% in the age groups from 20–59 years old. The total percentage of anti-HAV among the Japanese population was 16.40%. The results are summarized in Table 1 The estimation of anti-HAV positives among general population aged 20–79 in Japan was 15, 381, 558 (95%CI: 12, 000, 399–20, 030, 039).

**Table 1:** Estimated number of anti-HAV positives among general population aged 20–79 in Japan.

	Population in 2015 Census	Prevalence of anti-HAV in study subjects [95%CI]		Estimated Number of anti-HAV positives in Japan [95%CI]	
**Male**					
20–29 y.o.	6, 224, 913	0.00%	[0.0–3.7%]	0	[0–230, 322]
30–39 y.o.	7, 843, 971	1.00%	[0.0–3.0%]	78, 439	[0–343, 647]
40–49 y.o.	9, 287, 757	0.00%	[0.0–3.7%]	0	[0–230, 322]
50–59 y.o.	7, 731, 854	2.00%	[0.0–4.7%]	154, 637	[0–363, 397]
60–69 y.o.	8, 880, 633	28.00%	[19.2–36.8%]	2, 486, 577	[1, 705, 082–3, 268, 073]
70–79 y.o.	6, 415, 400	70.00%	[61.0–79.0%]	4, 490, 780	[3, 913, 394–5, 068, 166]
**Female**					
20–29 y.o.	5, 951, 429	0.00%	[0.0–3.7%]	0	[0–220, 203]
30–39 y.o.	7, 578, 404	2.00%	[0.0–4.7%]	151, 568	[0–356, 185]
40–49 y.o.	9, 017, 790	0.00%	[0.0–3.7%]	0	[0–333, 658]
50–59 y.o.	7, 696, 954	2.00%	[0.0–4.7%]	153, 939	[0–361, 757]
60–69 y.o.	9, 319, 173	26.00%	[17.4–34.6%]	2, 422, 985	[1, 621, 536–3, 224, 434]
70–79 y.o.	7, 665, 680	71.00%	[62.1–79.9%]	5, 442, 633	[4, 760, 387–6, 124, 878]
Total	93, 613, 958	16.40%	[12.8–21.4%]	15, 381, 558	[12, 000, 399–20, 030, 039]

## Discussion

HAV particles were first discovered in stool specimens of acute hepatitis cases using immunoelectron microscopy in 1973^15^. HAV exists in a lipid-enveloped (LE) form in human plasma^16^, this virus is 27 nm long, spherical particle containing a linear, single-stranded, and positive-sense RNA genome classified in the genus *Hepatovirus* of the family *Picornaviridae*, which remains infectious for significant periods on surfaces, in the environment, and in uncooked food^8^. Infection may result in asymptomatic or mild to severe acute hepatitis after an incubation period of 14–28 days. Complications and fatal outcomes are higher in older age groups, with the severity of disease increasing with age and relapse can also occur. However, lifelong immunity occurs once a person has been infected with HAV^1^.

In fact, HAV can be found in feces of infected persons because it is resistent to bile and protease released from digestive tract. Once outside the body, HAV can be inactivated at a temperature of 85 degrees for 1–2 minutes^17^.

In fact, the majority of HAV infection is transmitted via the fecal-oral route, either by personal contact or ingestion of food and water contaminated with HAV. Therefore, HAV is closely associated with a country’s socio-economic status and its prevalence varies region by region, from very low prevalence in countries with good sanitation and personal hygiene to very high prevalence in countries with poor or inadequate sanitation and personal hygiene. Over the last two decades, improvement in sanitation and personal hygiene has caused a shift in anti-HAV sero-prevalence in almost all countries. As a result, many people are susceptible to infection highlighting the risk of outbreak in the case of deterioration in sanitation and personal hygiene^18^.

Almost all countries worldwide are now facing two different types of HAV-related public health concerns. The low prevalence means a low rate of transmission and low infectivity among the population. In developed countries, sanitation and hygienic conditions have improved through effective measures, such as health education, lifestyle changes, and proper hand washing. In the United States, the prevalence of HAV varies region by region, with a slight decline in the prevalence among children and young adults from 29.5% (95% confidence interval (CI); 28.0–31.1%) during 1999–2006 to 24.2% (95% CI; 22.5–25.9%) during 2007–2012^19^.

The WHO recommends inoculation for health care workers who have frequent contact opportunities with hepatitis A patients, those with underlying diseases without HAV antibodies, and men who have sex with men^20^. In United State, hepatitis A vaccine became available for children aged 12–23 months in 2005, allowing for its incorporation into the routine early childhood vaccination schedule^21^. On the other hand, a free universal HAV childhood vaccination is not provided in Japan.

In Korea, high anti-HAV prevalence of approximately 96% was reported in 1980, and an outbreak affecting a million people has occurred twice in China, in 1983 and 1988. Therefore, Korea and China introduced the HAV vaccine for high risk populations in 1997^22^ and 2007^23^, respectively, after which it was included in the national immunization program starting from 2007 for China and 2015 for Korea. As a result, the level of active immunity to HAV via the vaccine increased dramatically. The decision to implement the HAV vaccine program in particular countries may depend on the extent of the disease as a public health problem, and whether benefits outweigh the cost for implementation.

Japan is a developed country that has improved its sanitation and personal hygiene since the late 1900s. The first nationwide sero-prevalence study was conducted in 1973, showing very low prevalence in those under 30 years of age and a prevalence as high as 96.9% in individuals older than 50 years. The second time nationwide sero-prevalence study was conducted in 1984, in which very low prevalence was found before the age of 40 and the prevalence for those over 50 years of age was maintained. In the third and fourth studies, conducted in 1994 and 2003, the occurrence of very low sero-prevalence shifted to ages under 50 years. The prevalence among the over-50 age group also decreased to 74.3% in 1994^14^ and 50.3% in 2003^13^.

The 2003 nationwide survey was the last study on anti-HAV sero-prevalence among the general population in Japan. According to the Infectious Agents Surveillance Report in 2014, 1,229 cases of HAV infection were reported during the period from 2010 to the 48th week of 2014 in Japan. Infections mostly occurred in males over 40 years of age. Out of 1,229 cases, 987 cases (80%) were suspected of oral infection and the presumed causative diet of 41% (405/987) was oyster and other seafood^24^.

In 1995, the HAV vaccine was marketed for individuals over 16 years of age in Japan, with use in individuals under 16 years of age approved in 2013. Currently, HAV vaccination is optional and inoculation is recommended only to those traveling to countries with  moderate to high prevalence of HAV.

Additionally, as a result of Japan internationalism, HAV can result from both foreign people entering Japan from HAV endemic areas and Japanese traveling to HAV endemic areas. Therefore, the prevention of HAV transmission due to internationalism required attention.

**Figure 2:**
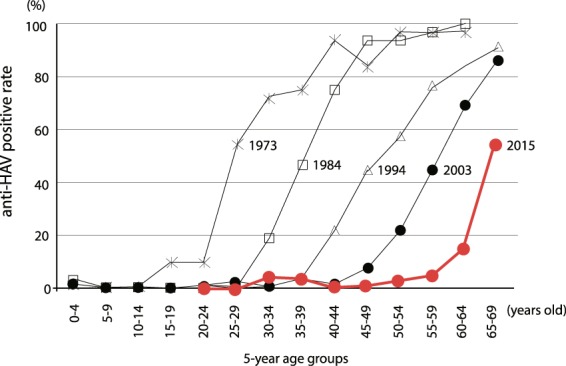
Age specific sero-prevalence of anti-HAV compared with four previous nationwide studies in Japan. The black line represents the transition of anti-HAV antibody positive rate measured by the National Institute of Infectious Diseases every 10 years and the red line represents the results of this study.

In this study, a similar pattern of age- and sex- specific sero-positivity of anti-HAV antibody was observed compared to the previous four nationwide studies conducted over four decades (Fig. 2). The curve for anti-HAV in this study also showed two different phases. Initially, the curve was extremely linear and adjacent to the horizontal x-axis before the age of 50 years, showing the very low immunity to HAV among younger population. After 50 years of age, the curve dramatically raised to a peak of 70.5% in the over-70 age group, showing the increasing trend of anti-HAV prevalence among the elderly. It can be clearly stated that the cohort reduction sustainably occurred during these four consecutive decades and that this small study was consistent with the previous nationwide studies, highlighting the anti-HAV prevalence from 2013–2015 among the general population.

This cohort reduction trend of anti-HAV in Japan results in low immunity to HAV infection and increased susceptibility to the infection, especially in the younger population, exposing the high possibility of mass outbreak in the future. As sanitation and personal hygiene have improved alongside economic development, HAV is less likely to be a public health concern. However, it is important to note that the younger population has no immunity to HAV in the case of an outbreak and an emergency response should be considered.

In conclusion, awareness campaigns regarding the possible outbreak of HAV in Japan and its preventive measures should be accelerated. The available HAV vaccine might be introduced to the general population, especially to younger generations, and high risk populations who are exposed to the source of infection, such as frequent travelers to HAV endemic areas, medical professionals who have contact with infected persons, anti-HAV negative patients with chronic liver disease,  and men who have sex with men.

## Methods

### Samples

A total of 1,200 serum samples were randomly selected from 7,682 stocked serum samples, all of which were obtained from annual health check-ups of residents and employees in Hiroshima prefecture during 2013–2015. In Hiroshima, the prefectural health medical promotion organization provides annual health check-ups to residents aged over 40 and employees of any age.

Assuming a 2% expected positive rate for all age groups and a 2% absolute accuracy, the sample size was calculated as 1.96^2^ × (0.02) × (1–0.02)/0.02^2^ = 188.2^25^. 100 people were calculated for each age class with a ratio of 1: 1 for both male and female. Therefore, a sample size of 200 was required.

Of the total 1,200 serum samples selected, 100 serum samples from both males and females for each specific age group (20–29, 30–39, 40–49, 50–59, 60–69, and 70–79) were used for subgroup analysis.

All serum samples were stored at −25 °C until serological measurement. Total anti-HAV was determined for all recruited serum samples using Lumipulse^®^ II HAV-Ab (Fujirebio, Inc., Tokyo, Japan). All anti-HAV were detected by Chemiluminescent Enzyme Immunoassay (CLEIA).

The overall HAV prevalence was estimated based on the number of anti-HAV positive samples among the total number of recruited serum samples, after which subgroup analysis was performed for each sex and age group.

Then, the age- and sex-specific anti-HAV positive prevalence in Hiroshima was calculated. Age- and sex- specific subpopulation numbers were obtained from population census of  Japanese. The prevalence and subpopulation were multiplied to estimate the total number of anti-HAV positive individuals as the whole Japan.

### Ethical consideration

This study was approved by Epidemiological Research Ethic Review Committee of Hiroshima University (Hiroshima University, E-3). All participants provided written informed consent. All methods were performed in accordance with the relevant guidelines and regulations.
